# Use of Cannabis and Other Pain Treatments Among Adults With Chronic Pain in US States With Medical Cannabis Programs

**DOI:** 10.1001/jamanetworkopen.2022.49797

**Published:** 2023-01-03

**Authors:** Mark C. Bicket, Elizabeth M. Stone, Emma E. McGinty

**Affiliations:** Department of Anesthesiology, University of Michigan, Ann Arbor; Department of Health Policy and Management, Johns Hopkins Bloomberg School of Public Health, Baltimore, Maryland; Department of Health Policy and Management, Johns Hopkins Bloomberg School of Public Health, Baltimore, Maryland

## Introduction

Most states have enacted laws allowing individuals to treat chronic pain with cannabis.^1^ Evidence is mixed about whether medical cannabis serves as a substitute for prescription opioids or other pain treatments.^2,3^ Accurate estimates of cannabis use or its substitution in place of pain treatments among adults with chronic noncancer pain are, to our knowledge, not available.^4^

## Methods

In this cross-sectional study, we surveyed a representative sample of adults aged 18 years or older with chronic pain who lived in the 36 states (and Washington, DC) with active medical cannabis programs in March to April 2022 (eTable 1 in Supplement 1). We fielded the survey using the National Opinion Research Center (NORC) AmeriSpeak panel. This probability-based panel includes about 54 000 members and is sourced from a sample covering 96% of US households with a recruitment rate of 34%.^5^ We defined chronic noncancer pain using the National Health Interview Survey (NHIS) criterion of pain unrelated to cancer on most days or every day in the past 6 months.^6^ The survey was conducted from March 3, 2022, to April 11, 2022. A screener survey consented and identified people with chronic pain (response rate: 75.4%), who were invited to complete the full survey (eTable 2 in Supplement 1).

We assessed self-reported use (ever, past 12 months, past 30 days) of medical cannabis, pharmacologic treatments (prescription opioids, prescription nonopioid analgesics, and over-the-counter analgesics), common nonpharmacologic treatments (physical therapy, meditation, cognitive behavioral therapy), and substitution of cannabis in place of these treatments for chronic pain. All analyses incorporated survey sampling weights to generate estimates representative of the 36 included states and Washington, D.C. The investigation was approved by the Johns Hopkins Bloomberg School of Public Health institutional review board and followed the STROBE reporting guideline for cross-sectional studies. Statistical analysis was performed using Stata statistical software version 15 (StataCorp).

## Results

Of the 1724 individuals identified as having chronic pain, 1661 (96.3%) completed the full survey (948 [57.1%] female; mean [SD] age, 52.3 [16.9] years); 31.0% (95% CI, 28.2%-34.1%) of adults with chronic pain reported having ever used cannabis to manage their pain; 25.9% (95% CI, 23.2%-28.8%) reported using cannabis to manage their chronic pain in the past 12 months, and 23.2% (95% CI, 20.6%-26.0%) reported using cannabis in the past 30 days. Most people who reported using cannabis to manage chronic pain also reported having used either at least 1 other pharmacologic (94.7%; 95% CI, 91.3%-96.8%) or nonpharmacologic pain treatment (70.6%; 95% CI, 64.8%-75.7%).

**Figure 1.**
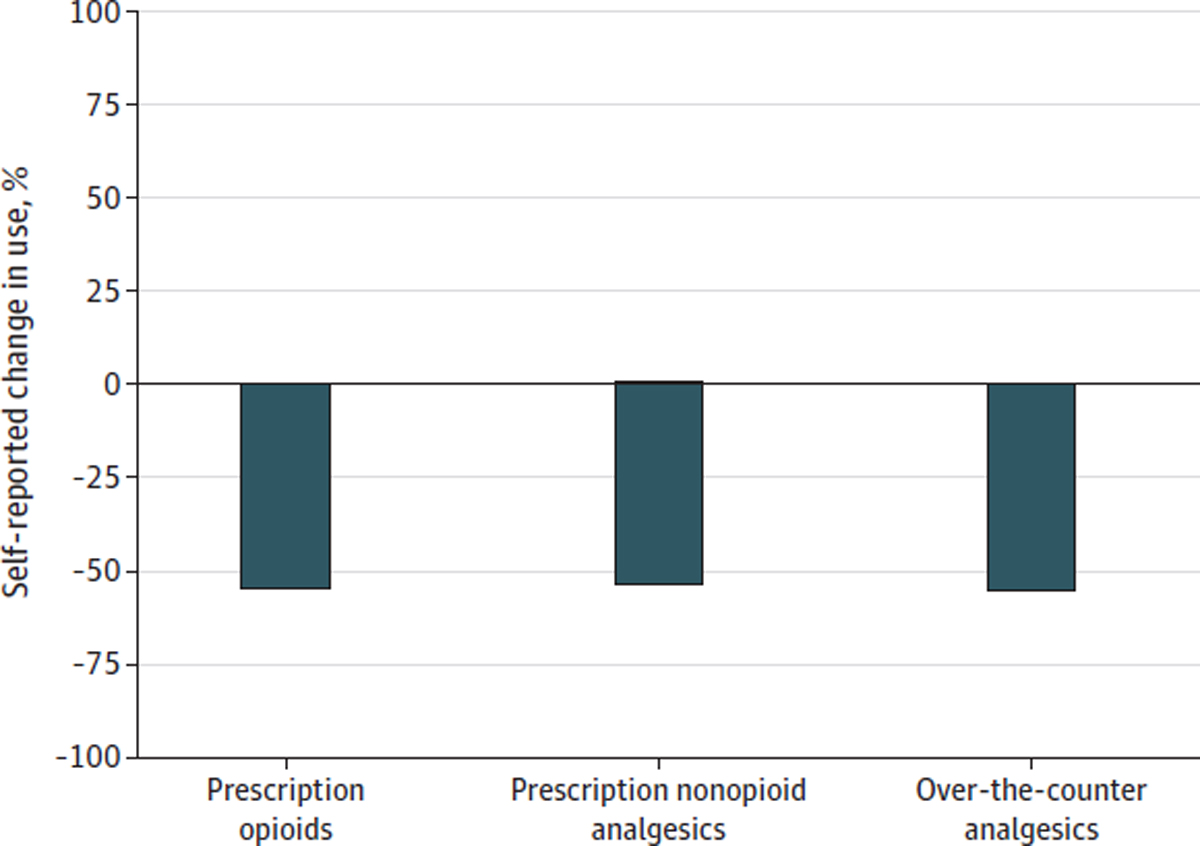
Self-reported Change in the Use of Pain Medications Due to Cannabis Use Among US Adults Aged 18 Years or Older With Chronic Pain in March to April 2022 Measures for pharmacologic pain treatments were from a survey fielded from March 3, 2022, to April 11, 2022, of adults aged at least 18 years living in states with medical cannabis laws who reported having chronic noncancer pain (n = 1661), used cannabis at any time (n = 495), and who also used pharmacologic treatments for pain (n = 474)). Respondents were asked “Has your use of cannabis to manage your chronic pain changed your use of any of the below?” with possible responses of increased, decreased, or no change. Bars signify the weighted proportion reporting increased use or decreased use.

**Figure 2.**
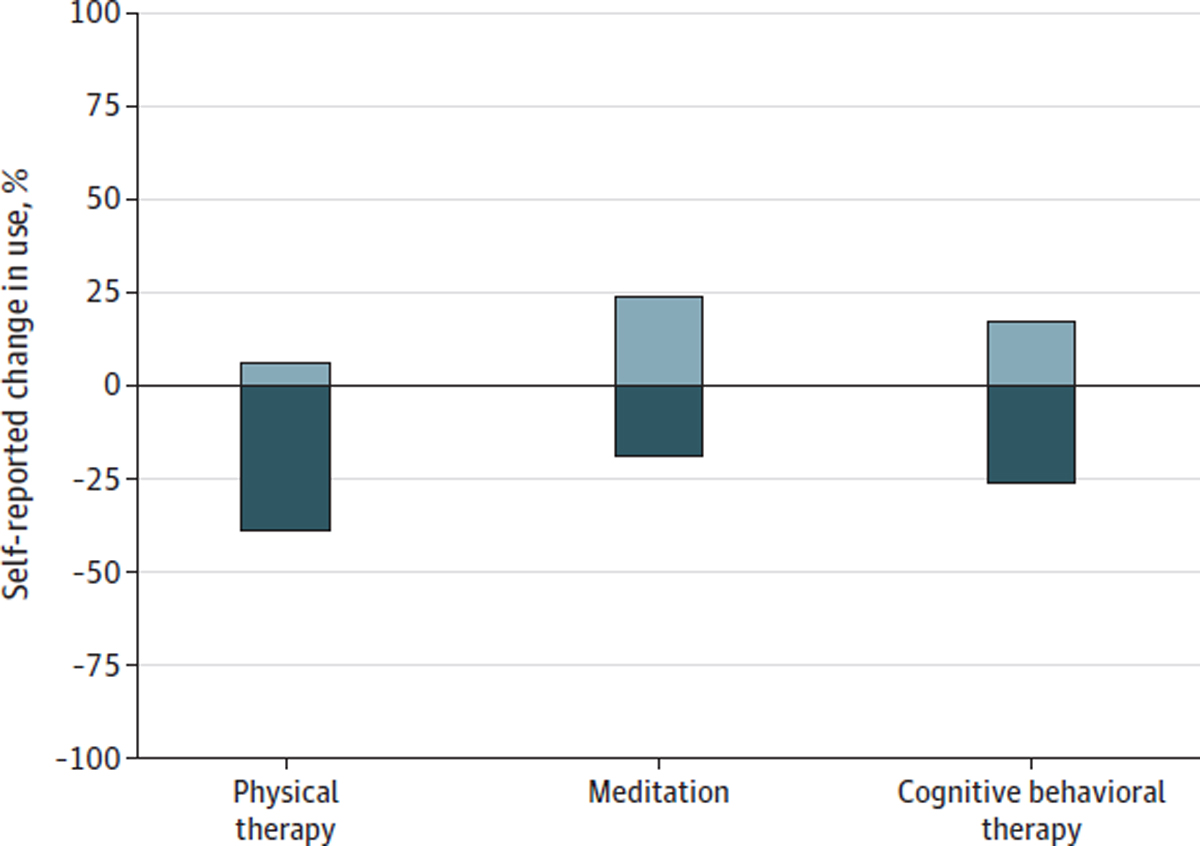
Self-reported Change in the Use of Common Nonpharmacologic Pain Treatments Due to Cannabis Use Among US Adults Aged 18 Years or Older With Chronic Pain in March to April 2022 Measures for nonpharmacologic pain treatments were from a survey fielded from March 3, 2022, to April 11, 2022, of adults aged at least 18 years living in states with medical cannabis laws who reported having chronic noncancer pain (n = 1661), used cannabis at any time (n = 495), and who used nonpharmacologic treatments for pain (n = 362). Respondents were asked “Has your use of cannabis to manage your chronic pain changed your use of any of the below?” with possible responses of increased, decreased, or no change. Bars signify the weighted proportion reporting an increased use or decreased use.

More than half of adults who used cannabis to manage their chronic pain reported that use of cannabis led them to decrease use of prescription opioid, prescription nonopioid, and over-the-counter pain medications, and less than 1% reported that use of cannabis increased their use of these medications (Figure 1). Fewer than half of respondents reported that cannabis use changed their use of nonpharmacologic pain treatments. Among adults with chronic pain in this study, 38.7% reported that their used of cannabis led to decreased use of physical therapy (5.9% reported it led to increased use), 19.1% reported it led to decreased use of meditation (23.7% reported it led to increased use), and 26.0% reported it led to decreased used of cognitive behavioral therapy (17.1% reported it led to increased use) (Figure 2).

## Discussion

Among adults with chronic pain in states with medical cannabis laws, 3 in 10 persons reported using cannabis to manage their pain. Most persons who used cannabis as a treatment for chronic pain reported substituting cannabis in place of other pain medications including prescription opioids. The high degree of substitution of cannabis with both opioid and nonopioid treatment emphasizes the importance of research to clarify the effectiveness and potential adverse consequences of cannabis for chronic pain. Our results suggest that state cannabis laws have enabled access to cannabis as an analgesic treatment despite knowledge gaps in use as a medical treatment for pain. Limitations include the possibility of sampling and self-reporting biases, although NORC AmeriSpeak uses best-practice probability-based recruitment,^5^ and changes in pain treatment from other factors (eg, forced opioid tapering).

## Supplementary Material

Supplement 2 Data Sharing Statement

Supplement 1 **eTable 1.** List of States and Districts with Active Medical Cannabis Programs as of March 2022
**eTable 2.** List of Survey Questions

## Data Availability

See Supplement 2.

## References

[1] Tormohlen KN, Bicket MC, White S, The state of the evidence on the association between state cannabis laws and opioid-related outcomes: A review. Curr Addict Rep. 2021;8(4):538–545. Published online September 28, 2021. doi:10.1007/s40429-021-00397-1 35668861 PMC9164259

[2] National Academies of Sciences, Engineering, and Medicine; Health and Medicine Division; Board on Health Sciences Policy; Phillips JK, Ford MA, Bonnie RJ, , eds. Pain Management and the Opioid Epidemic: Balancing Societal and Individual Benefits and Risks of Prescription Opioid Use. National Academies Press (US); 2017. 29023083

[3] Campbell G, Hall WD, Peacock A, Effect of cannabis use in people with chronic non-cancer pain prescribed opioids: findings from a 4-year prospective cohort study. Lancet Public Health. 2018;3(7):e341–e350. doi:10.1016/S2468-2667(18)30110-5 29976328 PMC6684473

[4] National Academies of Sciences, Engineering, and Medicine; Health and Medicine Division; Board on Population Health and Public Health Practice; Committee on the Health Effects of Marijuana: An Evidence Review and Research Agenda. The health effects of cannabis and cannabinoids: the current state of evidence and recommendations for research. Published January 12, 2017. Accessed November 30, 2022. https://www.ncbi.nlm.nih.gov/books/NBK423845/

[5] NORC AmeriSpeak. Technical overview of the AmeriSpeak Panel: NORC’s probability-based household panel. Updated February 22, 2022. June 17, 2022. https://amerispeak.norc.org/content/dam/amerispeak/research/pdf/AmeriSpeak%20Technical%20Overview%202019%2002%2018.pdf

[6] Dahlhamer J, Lucas J, Zelaya C, Prevalence of chronic pain and high-impact chronic pain among adults -United States, 2016. MMWR Morb Mortal Wkly Rep. 2018;67(36):1001–1006. doi:10.15585/mmwr.mm6736a2 30212442 PMC6146950

