# Supplementary material for: Use of Cannabis and Other Pain Treatments Among Adults With Chronic Pain in US States With Medical Cannabis Programs
Source: JAMA Netw Open. Author manuscript; Available in PMC 2024 Jan 3. (PMC9857553; doi:10.1001/jamanetworkopen.2022.49797)
Supplement: Supplement 2 — Data Sharing Statement [file NIHMS1865908-supplement-Supplement_2.pdf]

Data Sharing Statement

Bicket. Use of Cannabis and Other Pain Treatments Among Adults With Chronic Pain in US States With Medical Cannabis Programs. *JAMA Netw Open*. Published January 06, 2023. doi:10.1001/jamanetworkopen.2022.49797

Data
Data available: No

Additional Information
Explanation for why data not available: The data will not be shared per the data use agreement with NORC, which only allows access by the study team.
