# Supplementary material for: Use of Cannabis and Other Pain Treatments Among Adults With Chronic Pain in US States With Medical Cannabis Programs
Source: JAMA Netw Open. Author manuscript; Available in PMC 2024 Jan 3. (PMC9857553; doi:10.1001/jamanetworkopen.2022.49797)
Supplement: Supplement 1 — eTable 1. List of States and Districts with Active Medical Cannabis Programs as of March 2022 eTable 2. List of Survey Questions [file NIHMS1865908-supplement-Supplement_1.pdf]

Supplemental Online Content

Bicket MC, Stone EM, McGinty EE. Use of cannabis and other pain treatments among adults with chronic pain in US states with medical cannabis programs. *JAMA Netw Open.* 2023;6(1):e2249797. doi:10.1001/jamanetworkopen.2022.49797

eTable 1. List of States and Districts with Active Medical Cannabis Programs as of March 2022

eTable 2. List of Survey Questions

This supplemental material has been provided by the authors to give readers additional information about their work.

© 2023 Bicket MC et al. *JAMA Network Open.*

eTable 1. List of States and Districts with Active Medical Cannabis Programs as of March 2022

Alaska
Alabama
Arkansas
Arizona
California
Colorado
Connecticut
District of Columbia
Delaware
Florida
Hawaii
Illinois
Louisiana
Massachusetts
Maryland
Maine
Michigan
Minnesota
Missouri
Montana
North Dakota
New Hampshire
New Jersey
New Mexico
Nevada
New York
Oregon
Ohio
Oklahoma
Pennsylvania
Rhode Island
South Dakota
Utah
Virginia
Vermont
Washington
West Virginia

© 2023 Bicket MC et al. *JAMA Network Open.*

eTable 2. List of Survey Questions

Question	Response options
In the past 6 months, how often did you have pain? Would you say never, some days, most days, or every day?	Never<br>Some days<br>Most days<br>Every day<br>DON'T KNOW
Is your pain related to cancer?	Yes<br>No<br>DON'T KNOW
Do you still reside in [INSERT [STATE]]?	Yes<br>No<br>DON'T KNOW
If no, what state do you reside in?	DON'T KNOW<br>Alaska<br>Alabama<br>Arkansas<br>Arizona<br>California<br>Colorado<br>Connecticut<br>District of Columbia<br>Delaware<br>Florida<br>Georgia<br>Hawaii<br>Iowa<br>Idaho<br>Illinois<br>Indiana<br>Kansas<br>Kentucky<br>Louisiana<br>Massachusetts<br>Maryland<br>Maine<br>Michigan<br>Minnesota<br>Missouri<br>Mississippi<br>Montana<br>North Carolina<br>North Dakota<br>Nebraska<br>New Hampshire<br>New Jersey<br>New Mexico<br>Nevada<br>New York

© 2023 Bicket MC et al. *JAMA Network Open.*

	Oregon<br>Ohio<br>Oklahoma<br>Pennsylvania<br>Rhode Island<br>South Carolina<br>South Dakota<br>Tennessee<br>Texas<br>Utah<br>Virginia<br>Vermont<br>Washington<br>Wisconsin<br>West Virginia<br>Wyoming
Would you be willing to complete this survey?	I agree to participate<br>I do not agree to participate<br>DON'T KNOW
Have you ever used cannabis to manage your chronic pain?	Yes<br>No<br>DON'T KNOW
Have you used cannabis to manage your chronic pain in the past 12 months?	Yes<br>No<br>DON'T KNOW
Have you used cannabis to manage your chronic pain in the past 30 days?	Yes<br>No<br>DON'T KNOW
Have you ever used any of the following non-medication approaches to manage your chronic pain?<br><br>Items:<br>Surgery<br>Injections<br>Nerve block<br>Physical therapy<br>Yoga<br>Massage<br>Meditation<br>Cognitive behavioral therapy<br>Acupuncture<br>Heat, hot packs, or other heat therapies<br>Cold, cryotherapy, or other cold therapies<br>Biofeedback or relaxation therapy<br>Transcutaneous electrical nerve stimulation (TENS)<br>Acupressure<br>Exercise<br>Aquatic/pool therapy	For each item:<br><br>Yes, in the past 30 days<br>Yes, in the past 12 months<br>Yes, not in the past 12 months<br>No

© 2023 Bicket MC et al. *JAMA Network Open.*

Chiropractic therapy<br>Cupping therapy<br>Other non-medication strategy to manage chronic pain (please describe)	
Has your use of cannabis to manage your chronic pain changed your use of any of the below?<br><br>Items [For each treatment populated in prior question]:<br>Surgery<br>Injections<br>Nerve block<br>Physical therapy<br>Yoga<br>Massage<br>Meditation<br>Cognitive behavioral therapy<br>Acupuncture<br>Heat, hot packs, or other heat therapies<br>Cold, cryotherapy, or other cold therapies<br>Biofeedback or relaxation therapy<br>Transcutaneous electrical nerve stimulation (TENS)<br>Acupressure<br>Exercise<br>Aquatic/pool therapy<br>Chiropractic therapy<br>Cupping therapy<br>Other non-medication strategy to manage chronic pain (please describe)	Yes, increased use of [item]<br>Yes, decreased use of [item]<br>No<br>Prefer not to answer

© 2023 Bicket MC et al. *JAMA Network Open.*
